# CT引导下经皮肺穿刺活检术并发症发生的影响因素分析

**DOI:** 10.3779/j.issn.1009-3419.2024.101.03

**Published:** 2024-03-20

**Authors:** Xing WANG, Hong ZHANG, Xun ZHANG

**Affiliations:** ^1^300051 天津，天津市胸科医院影像科; ^1^Department of Radiology, Tianjin Chest Hospital, Tianjin 300051, China; ^2^300051 天津，天津市胸科医院胸外科; ^2^Department of Thoracic Surgery, Tianjin Chest Hospital, Tianjin 300051, China

**Keywords:** 肺肿瘤, 活检, 计算机断层扫描, 气胸, 肺出血, Lung neoplasms, Biopsy, Computed tomography, Pneumothorax, Pulmonary hemorrhage

## Abstract

**背景与目的:**

计算机断层扫描引导下经皮肺穿刺活检术（computed tomography guided percutaneous lung biopsy, CT-PLB）是目前临床广泛应用的肺部病变诊断方式，但其为有创检查，最常见的并发症为气胸和肺出血，严重时可危及生命。本研究旨在分析影响CT-PLB不同并发症发生的独立危险因素，以降低并发症发生率。

**方法:**

回顾性分析2018年5月至2019年12月于我院行CT-PLB且临床资料完整的605例患者的资料。依病变位置分为胸膜下组和非胸膜下组，根据并发症分为气胸组、肺出血组、气胸合并肺出血组及无气胸/肺出血组，计算各并发症的发生率。分析影响不同并发症发生的危险因素及各并发症的独立危险因素。

**结果:**

气胸发生率为34.1%，肺出血发生率为28.1%，同时发生气胸及肺出血63例，发生率为10.8%。影响胸膜下组气胸发生的独立危险因素为病变大小（P=0.002）。影响非胸膜下组气胸发生的独立危险因素为穿刺针走行区域平扫CT值（P=0.035）、穿刺针经过肺组织长度（P=0.003）、穿刺针经过胸壁厚度（P=0.020）；影响非胸膜下组肺出血发生的独立危险因素为穿刺针经过肺组织长度（P<0.001）、穿刺针走行区域△CT值（P=0.001）、病变大小（P=0.034）及患者体位（P=0.014）。影响气胸、肺出血同时发生的独立危险因素为穿刺针经过肺组织长度（P<0.001）、穿刺针走行区域△CT值（P<0.001）。

**结论:**

CT-PLB是一种安全、有效的诊断方式，对肺部占位性病变具有较高的诊断价值，选择合适的穿刺方案可减少气胸、肺出血等并发症，提高诊疗效率。

## Abstract (en)

**Background and objective:**

Computed tomography guided percutaneous lung biopsy (CT-PLB) is a widely used method for the diagnosis of lung lesions. However, it is invasive, and the most common complications are pneumothorax and pulmonary hemorrhage, which can be life-threatening in severe cases. Therefore, the aim of this study is to analyze the independent risk factors affecting the occurrence of different complications of CT-PLB, so as to reduce the incidence of complications.

**Methods:**

The 605 patients with complete clinical data who underwent CT-PLB in our hospital from May 2018 to December 2019 were retrospectively analyzed. According to the location of the lesions, they were divided into subpleural group and non-subpleural group. The patients were divided into pneumothorax group, pulmonary hemorrhage group, pneumothorax with pulmonary hemorrhage group and non-pneumothorax/pulmonary hemorrhage group according to the complications. The risk factors affecting the incidence of different complications and the independent risk factors of each complication were analyzed.

**Results:**

The incidence of pneumothorax was 34.1%, the incidence of pulmonary hemorrhage was 28.1%, and the incidence of pneumothorax complicated with pulmonary hemorrhage was 10.8% (63 cases). The independent risk factor affecting the incidence of subpleural pneumothorax was lesion size (P=0.002). The independent risk factors affecting the occurrence of pneumothorax in the non-subpleural group were plain scan CT value (P=0.035), length of needle through lung tissue (P=0.003), and thickness of needle through chest wall (P=0.020). Independent risk factors affecting the occurrence of pulmonary hemorrhage in the non-subpleural group were length of needle through lung tissue (P<0.001), △CT value of needle travel area (P=0.001), lesion size (P=0.034) and body position (P=0.014). The independent risk factors affecting the co-occurrence of pneumothorax and pulmonary hemorrhage were the length of needle through lung tissue (P<0.001) and the △CT value of needle travel area (P<0.001).

**Conclusion:**

CT-PLB is a safe and effective diagnostic method, which of high diagnostic value for lung lesions. Selecting the appropriate puncture program can reduce complications such as pneumothorax and pulmonary hemorrhage, and improve diagnosis and treatment efficiency.

2020年全球癌症统计数据^[1]^表明：肺癌发病率位居恶性肿瘤的第二位，是癌症死亡的最主要原因。根据国家癌症登记处的数据^[2]^，肺癌仍然是我国发病率和死亡率最高的癌症。肺癌高死亡率的主要原因是早期多无特异性临床症状，当出现咳嗽、咯血等症状就诊时常已处于晚期，可选择的治疗方式有限，导致患者生存期短、预后较差。肺癌治疗方式的选择和预后与临床分期密切相关，因此早诊断、早治疗是改善其预后的关键。筛查是早期发现癌症和癌前病变的重要方法，随着低剂量胸部计算机断层扫描（computed tomography, CT）在肺癌筛查中的广泛使用，越来越多的肺部结节被发现^[3]^，直径>10 mm的结节，甚至可能具有较高生长率的更小结节，都需要密切随访，甚至行CT引导下经皮肺穿刺活检术（CT-guided percutaneous lung biopsy, CT-PLB）^[4]^。

CT-PLB是当前被广泛接受的肺部病变诊断方式，在肺癌的诊断和治疗中起着越来越重要的作用。但其为有创检查，最常见的并发症为气胸和肺出血^[5]^。既往文献^[6]^报道与气胸发生相关的危险因素包括：慢性阻塞性肺疾病的存在、无同侧肺部手术史、病变小、穿刺路径长和反复胸膜穿刺；与肺出血相关的危险因素主要有切割针的大小、病变大小、穿刺路径长短、是否存在肺动脉高压等。本研究根据病变位置和并发症进行分组，并引入新的相关危险因素，进一步分析影响CT-PLB后气胸及肺出血发生的独立危险因素，以期为临床治疗提供指导。

## 1 资料与方法

### 1.1 研究对象

连续收取2018年5月至2019年12月于我院行CT-PLB且临床资料完整的患者资料。本研究经我院伦理委员会批准。

### 1.2 检查操作方法

采用德国SIEMENS公司16排螺旋CT机，穿刺活检针为普利塞半自动穿刺活检针，规格型号分别为：18 G×100 mm和18 G×150 mm。穿刺步骤：将患者根据术前拟定的穿刺体位置于扫描床上，暴露穿刺部位皮肤，进行胸部CT平扫及增强检查，在定位像上以病灶为中心，上下均超过病灶2 cm为扫描野，行螺旋CT扫描，根据重建图像进一步确认穿刺进针点和路径，于体表做好标记。常规消毒后铺无菌巾，于穿刺点部位用利多卡因逐层浸润麻醉。根据预定好的穿刺进针点及偏转角度缓慢进针。再次小范围CT扫描，观察穿刺针的角度和深度，最终使穿刺针成功到达病灶内。利用穿刺活检针切取2-3个组织条，以10%甲醛溶液固定，将穿刺针抽离后，喷涂至载玻片涂片，同时助手将敷贴贴于穿刺进针处皮肤并按压2-3 min。穿刺针抽离后患者平卧5 min，然后以5 mm层厚行全肺野低剂量CT扫描，观察穿刺针道周围及全肺野是否有出血、气胸及胸腔积液等并发症。患者穿刺术后24 h复查床旁胸部平片，以发现延迟气胸及肺出血等。

### 1.3 分组

根据病变位置，将其分为胸膜下组及非胸膜下组。紧贴胸膜、与胸膜间距离为0 cm定义为胸膜下病变，而非胸膜下病变则指除胸膜下病变以外的肺部病变。根据并发症有无及种类，分为气胸组、肺出血组、气胸合并肺出血组及无气胸/肺出血组。

### 1.4 观察指标

术中、术后观察患者有无咯血，术后5 min全肺野CT扫描观察有无气胸、肺出血，术后24 h内复查床旁胸片以发现延迟气胸及肺出血。肺出血定义为穿刺活检术后CT扫描穿刺针道周围出现磨玻璃密度影或新的实变影^[7]^。影响气胸及肺出血发生的变量包括患者相关因素：性别、年龄；病灶相关因素：病变大小（病灶最大层面长径与短径乘积）、病变位置（右肺上中叶/右肺下叶/左肺上叶/左肺下叶）、病理类型（良/恶性）、穿刺针走行区域平扫CT值、穿刺针走行区域增强平均CT值（动脉期与静脉期CT值的平均值）、穿刺针走行区域△CT值（增强平均CT值-平扫CT值）；操作技术相关因素：患者体位（仰卧位/俯卧位/左侧卧位/右侧卧位）、穿刺针经过肺组织长度（病变深度）、穿刺针经过胸壁厚度、穿刺针长度（长针/短针）。相关参数测量方法见图1-3。

**图1:**
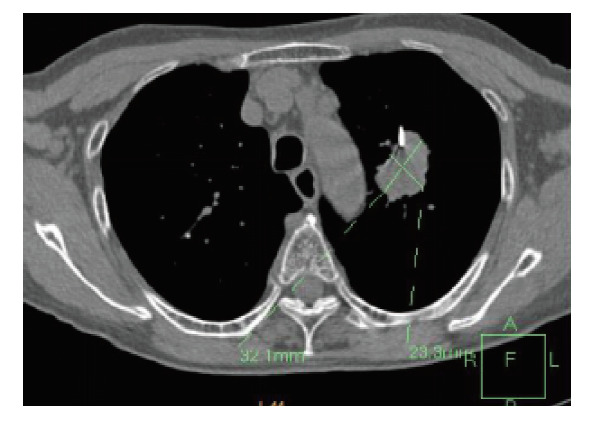
病变大小测量：穿刺层面病变长径×短径为3.21 cm×2.33 cm≈7.48 cm^2^

**图2:**
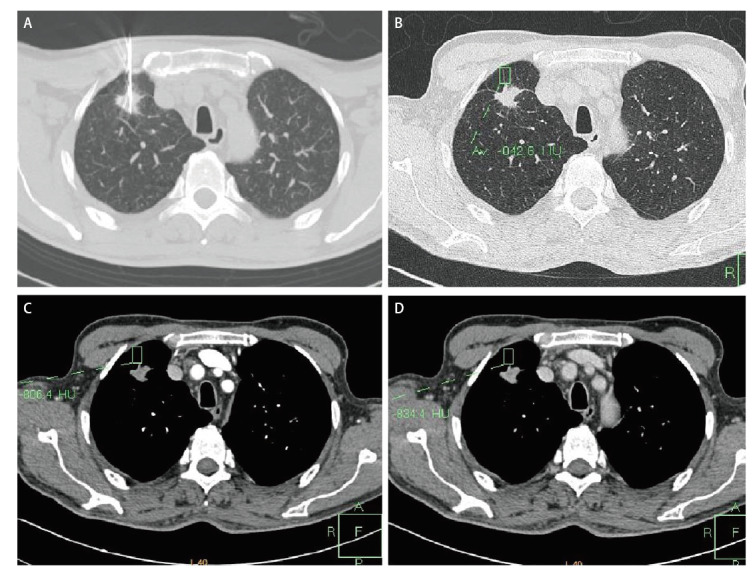
穿刺针走行区域CT值测量。A：穿刺针走行；B：穿刺针走行区域平扫CT值；C：穿刺针走行区域动脉期增强CT值；D：穿刺针走行区域静脉期增强CT值。

**图3:**
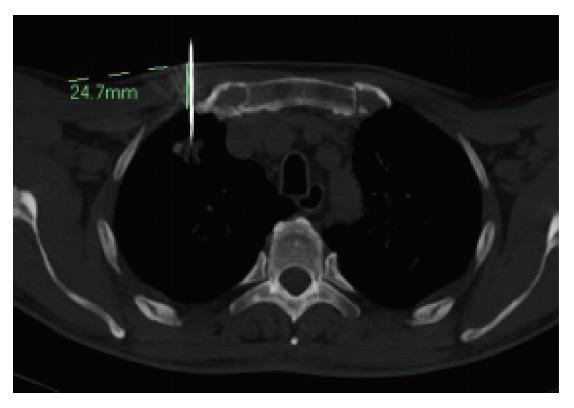
胸壁厚度测量：穿刺针与皮肤表面接触点到壁层胸膜之间距离

### 1.5 统计学方法

采用SPSS 23.0分析数据，计数资料以例（%）表示，组间比较采用χ^2^检验及Fisher’s确切概率法；符合正态分布的计量资料以均数±标准差（Mean±SD）表示，组间比较采用t检验，不符合正态分布的计量资料以中位数（四分位数）[M(Q1, Q3)]表示，组间比较采用非参数检验，均以P<0.05为差异有统计学意义。将组间比较有显著差异的变量纳入Logistic多变量回归分析模型，得出各并发症的独立危险因素，并计算回归系数及比值比（odds ratio, OR）。

## 2 结果

最终共605例患者被纳入本研究，其中男性371例，女性234例。年龄22-94（64.89±22.06）岁。21例因中途无法配合完成检查而排除，穿刺并取材成功584例，其中胸膜下组306例，非胸膜下组278例。气胸发生率为34.1%（199/584），肺出血发生率为28.1%（164/584），气胸合并肺出血发生率为10.8%（63/584）。胸膜下组气胸发生率为27.8%（85/306），因胸膜下组病变穿刺针不经过肺组织，故无肺出血发生。非胸膜下组气胸发生率为18.3%（51/278），肺出血发生率为36.3%（101/278），气胸合并肺出血63例，发生率为22.7%（63/278）。

### 2.1 胸膜下组结果

气胸组与无气胸/肺出血组比较，病变大小（P<0.001）、病变位置（P=0.017）组间比较差异有统计学意义（表1）；影响气胸发生的独立危险因素为病变大小（表2）。

**表1:** 胸膜下气胸组与无气胸/肺出血组各相关因素比较

Factors	Subpleural pneumothorax group (n=85)	Non-pneumothorax/pulmonary hemorrhage group (n=221)	P
Age (yr)	63.01±12.32	63.48±12.40	0.767
Gender (Male/Female)	51/34	149/72	0.222
Pathology (Benign/Malignant)	37/48	75/146	0.119
Lesion location (Right middle upper/Right lower/Left upper/ Left lower)	28/28/7/22	61/56/54/50	0.017
Body postion (Supine/Prone/Left side/Right side)	25/51/4/5	70/130/7/14	0.910
The thickness of needle across chest wall (mm)	29.13±8.84	30.46±10.06	0.285
Lesion size (cm^2^)	11.02 (5.46, 16.62)	16.56 (10.35, 30.92)	<0.001

**表2:** 胸膜下组Logistic多元回归分析影响气胸发生率的因素

Factors	B	SE	Wald	df	Sig	Exp (B)	95%CI for EXP (B)	
							Lower	Upper
Lesion size	-0.030	0.010	9.976	1	0.002	0.970	0.952	0.989
Lesion location	-0.014	0.073	0.039	1	0.844	0.986	0.855	1.137

B: partial regression coefficient; SE: the standard error of partial regression coefficient; Wald: statistic; EXP: the odds ratio; Sig: P value; CI: confidence interval.

### 2.2 非胸膜下组结果

气胸组与无气胸/出血组比较，穿刺针经过胸壁厚度（P=0.014）、穿刺针经过肺组织长度（P=0.001）、穿刺针走行区域平扫CT值（P=0.005）、穿刺针走行区域增强平均CT值（P=0.014）组间比较差异有统计学意义（表3）。影响气胸发生率的独立危险因素为穿刺针走行区域平扫CT值、穿刺针经过肺组织长度、穿刺针经过胸壁厚度（表4）。肺出血组与无气胸/肺出血组比较，患者年龄（P=0.033）、体位（P=0.004）、穿刺针长度（P=0.017）、病变大小（P=0.012）、穿刺针经过肺组织长度（P<0.001）、穿刺针走行区域平扫CT值（P=0.032）及穿刺针走行区域△CT值（P<0.001）组间比较差异有统计学意义（表3）。影响肺出血发生的独立危险因素依次为：穿刺针经过肺组织长度、穿刺针走行区域△CT值、病变大小、患者体位（表5）。气胸合并肺出血组与无气胸/肺出血组比较，穿刺针长度（P=0.002）、病变大小（P=0.001）、穿刺针经过肺组织长度（P<0.001）、穿刺针走行区域平扫CT值（P=0.013）、穿刺针走行区域△CT值（P<0.001）组间比较差异有统计学意义（表3）。影响气胸、肺出血同时发生的独立危险因素为穿刺针经过肺组织长度、穿刺针走行区域△CT值（表6）。

**表3:** 非胸膜下气胸组、非胸膜下肺出血组、非胸膜下气胸并肺出血组与无气胸/肺出血组各相关因素比较

Factors	Non-subpleural pneumothorax group (n=51)	Non-subpleural pulmonary hemorrhage group (n=101)	Non-subpleural pneumothorax combined with pulmonary hemorrhage group (n=63)	Non-pneumothorax/pulmonary hemorrhage group (n=63)	^a^P	^b^P	^c^P
Age (yr)	65.86±12.36	60.43±12.19	62.78±11.06	63.81±7.95	0.308	0.033	0.549
Gender (Male/Female)	33/18	55/46	42/21	40/23	0.893	0.254	0.709
Pathology (Benign/Malignant)	8/43	25/76	11/52	14/49	0.379	0.711	0.503
Lesion location (Right middle upper/Right lower/Left upper/Left lower)	20/14/12/5	36/18/31/16	26/17/11/9	28/13/9/13	0.247	0.126	0.674
Body postion (Supine/Prone/Left side/Right side)	18/26/4/3	47/53/0/1	26/33/2/2	14/44/1/4	0.118	0.004	0.104
Needle length (Short needle/Long needle)	51/0	86/15	46/17	61/2	0.199	0.017	0.002
Needle across chest wall thickness (mm)	29.64±9.44	37.89±15.13	34.26±14.30	34.38±10.59	0.014	0.084	0.957
Needle across lung tissue length (mm)	18.93±8.41	25.69±12.75	31.52±15.73	13.94±7.72	0.001	<0.001	<0.001
Lesion size (cm^2^)	10.85 (4.62, 20.40)	7.31 (4.22, 13.64)	6.09 (3.42, 10.73)	9.61 (5.04, 19.74)	0.664	0.012	0.001
CT value of plain scan	-780.70±83.50	-765.00±78.74	-775.27±93.09	-739.67±63.03	0.005	0.032	0.013
Mean CT value of enhancement	-773.84±93.98	-739.65±85.34	-743.29±107.04	-735.46±61.86	0.014	0.736	0.616
△CT value	3.45 (2.00, 6.95)	26.00 (14.20, 41.05)	26.20 (17.35, 36.05)	2.85 (1.15, 4.45)	0.509	<0.001	<0.001

a: non-subpleural pneumothorax group vs non-pneumothorax/pulmonary hemorrhage group; b: non-subpleural pulmonary hemorrhage group vs non-pneumothorax/pulmonary hemorrhage group; c: non-subpleural pneumothorax combined with pulmonary hemorrhage group vs non-pneumothorax/pulmonary hemorrhage group.

**表4:** 非胸膜下组Logistic多元回归分析影响气胸发生率的因素

Factors	B	SE	Wald	df	Sig	Exp (B)	95%CI for EXP (B)	
							Lower	Upper
Needle across chest wall thickness	-0.056	0.024	5.447	1	0.020	0.945	0.901	0.991
Needle across lung tissue length	0.086	0.029	8.607	1	0.003	1.088	1.028	1.150
CT value of plain scan	1.338	0.636	4.429	1	0.035	3.811	1.096	13.251
Mean CT value of enhancement	0.000	0.004	0.014	1	0.906	1.000	0.992	1.007

**表5:** 非胸膜下组Logistic多元回归分析影响肺出血发生率的因素

Factors	B	SE	Wald	df	Sig	Exp (B)	95%CI for EXP (B)	
							Lower	Upper
Age	-0.044	0.023	3.526	1	0.060	0.957	0.914	1.002
Needle across lung tissue length	0.157	0.035	19.879	1	<0.001	1.171	1.092	1.254
Lesion size	-0.053	0.025	4.474	1	0.034	0.949	0.904	0.996
CT value of plain scan	-0.003	0.004	0.823	1	0.364	0.997	0.990	1.004
△CT value	0.41	0.012	11.205	1	0.001	1.042	1.017	1.067
Patient postion	-1.000	0.407	6.029	1	0.014	0.368	0.166	0.817
Needle length	-0.710	0.978	0.527	1	0.468	0.492	0.072	3.342

**表6:** 胸膜下组Logistic多元回归分析影响气胸并肺出血发生率的因素

Factors	B	S.E.	Wald	df	Sig	Exp (B)	95%CI for EXP (B)	
							Lower	Upper
Needle across lung tissue length	0.136	0.034	15.641	1	<0.001	1.146	1.071	1.226
Lesion size	-0.053	0.033	2.603	1	0.107	0.949	0.890	1.011
CT value of plain scan	1.112	0.623	3.183	1	0.074	3.039	0.896	10.307
△CT value	0.076	0.020	13.811	1	<0.001	1.079	1.036	1.122
Needle length	-1.086	1.145	0.899	1	0.343	0.338	0.036	3.184

## 3 讨论

CT-PLB在肺部占位性病变的诊断中起着非常重要的作用，尤其适用于支气管镜检查受限的肺部外周病变。既往文献^[8]^报道肺气肿是气胸发生的独立危险因素，且肺气肿的存在与穿刺术后出现需要置管引流的气胸密切相关。本研究中我们引入了穿刺针走行区域平扫CT值，以此来反映穿刺路径上局部肺组织的气肿情况，同时为避免整体肺气肿情况对于局部气肿评价的影响，排除严重肺气肿、穿刺走行区存在气肿以及阻塞性肺气肿患者。同时我们还测量了穿刺针走行区域增强平均CT值及△CT值，增强平均CT值反映穿刺针走行区域的血管含量情况（包括动脉及静脉），△CT值为穿刺针走行区域增强平均CT值与平扫CT值之差，显示增强前后该区域内的CT值变化。胸壁软组织对穿刺针起到固定的作用，当胸壁厚度较小时，对穿刺针的固定作用较弱，操作难度增加，进而影响穿刺针与胸壁夹角的准确性；当胸壁较厚时，对穿刺针的固定作用过强，进针阻力及切割难度增加。

超声引导下肺穿刺活检术对肺部周围型病灶有独特的优势，超声引导下可以实时显示病灶部位、大小、血供情况，从而提高穿刺准确率。但超声引导下活检也有其局限性。首先，病灶位置只能局限在外周，而深部病灶超声无法显示，无法辅助穿刺。其次，较大体积的病灶内部可能含较多液化坏死组织，超声对其判断可能存在偏差，易穿刺到液化坏死组织，导致无法获得明确的病理诊断^[9]^。此外，纯磨玻璃病灶或混合性病灶在超声上显影不清，也会对穿刺效果产生影响。而胸部CT可直观地分辨病变的位置、大小、形态、密度及其毗邻关系，因此本研究病变均采用CT-PLB。倪颖梦等^[10]^将紧贴胸膜的肺部病变定义为病变深度0 cm，并发现病变深度为0 cm时，气胸和胸壁软组织积气的发生率为20.7%，明显低于非紧邻胸膜病变气胸和胸壁软组织积气的发生率（40%）。本研究胸膜下组气胸发生率为27.8%，与文献^[6,11]^报道的气胸发生率（19%-60%）相符合。既往文献^[8]^多以最大层面的最大径线定义病变大小，而肺部占位性病变形态多不规则，单用病变长径很难体现病变的真实大小，因此本研究以病变最大层面的长短径乘积作为衡量病变大小的指标，从而反映操作的难易程度。此外，胸膜下病变穿刺术后发生气胸，也间接说明了病变紧贴胸膜，发生脏层胸膜粘连的可能性大，而壁层胸膜受累概率低，进而为病变性质的判定提供间接证据^[12]^。

影响非胸膜下组气胸发生的独立危险因素包括：穿刺针走行区域平扫CT值、穿刺针经过肺组织长度及穿刺针经过胸壁厚度。穿刺针走行区域平扫CT值反映局部气肿情况，CT值越小，气肿程度越高，则发生气胸的概率越大，这与慢性阻塞性肺疾病及肺大疱是肺穿刺活检术后发生气胸的独立危险因素的理论^[13]^是一致的。与以往文献^[14]^报道类似，穿刺针经过肺组织长度，即病变深度是影响气胸发生的独立危险因素。当胸壁厚度较小时，胸壁对穿刺针的固定作用减弱，操作难度增加，进而增加并发症发生的风险。

穿刺针经过肺组织长度、病变大小是影响肺出血发生的独立危险因素^[15]^，病变深度增加导致穿刺针经过肺组织的长度增加，从而增加了穿刺路径上肺组织血管损伤的概率。病灶面积小会增加精确定位的难度，加上患者呼吸运动的影响，使得穿刺难度进一步加大，从而增加调整穿刺针角度的概率，增加穿刺所需时间，进而增加胸膜和肺损伤的机会^[15]^。我们首次提出穿刺针走行区域△CT值并进行测量统计，经多元回归分析发现其是肺出血的独立危险因素，△CT值反映穿刺针走行区域增强前、后CT值的变化，当该区域无明显病变时，△CT值可间接反映该区域局部血管含量，穿刺术后肺出血的发生，一部分就是因为穿刺针经过肺组织损伤肺小血管所致。该因素能较好地预测局部区域血管含量，指导术前穿刺路径的规划。仰卧位时患者呼吸运动幅度较大，穿刺难度增加，且仰卧体位时常选择前胸壁或侧胸壁经肋间入路，前胸壁及侧胸壁肌肉厚度不及背部，对穿刺针的固定作用小，这也是增加操作难度的另一个方面^[16]^。

气胸、肺出血同时发生较少，本组病例中发生率为10.8%，气胸和肺出血是肺穿刺活检术后最常见的两大并发症，既往文献^[8,15]^多单独分析影响气胸、肺出血发生的危险因素，而本研究中将两种并发症并存者单独分组，发现穿刺针经过肺组织长度及穿刺针走行区域△CT值为影响二者同时发生的独立危险因素。如前所述，穿刺针经过肺组织长度的增加会增加损伤胸膜和肺小血管的概率，从而增加气胸及肺出血发生的风险。穿刺针走行区域△CT值反映局部区域内血管含量，△CT值的升高会增加肺出血发生的概率，而其如何影响气胸的发生，推测在气胸、肺出血同时发生时，二者之间可能存在某种相互作用，有待后续进一步研究探索。

本研究存在一些不足。首先，未对肺出血按出血量进一步分级，以分析影响不同级别肺出血的危险因素是否存在差异；其次，未对气胸进一步细分，以分析影响术中/术后即刻气胸与延迟气胸的危险因素是否相同；最后，操作者相关因素（例如操作时间、穿刺活检经验等）也是影响并发症发生的重要因素，但由于不能细分量化，故未纳入该项研究。总之，CT-PLB是一种安全、有效的诊断方式，对于肺部占位性病变具有较高的诊断价值，选择合适的穿刺方案，有助于减少气胸、肺出血等并发症，提高诊疗效率。
